# A Methodology for Evaluating Organizational Change in Community-Based Chronic Disease Interventions

**Published:** 2007-09-15

**Authors:** Marilyn A Winkleby, Krista D Hanni, Elsa Mendoza, John Snider

**Affiliations:** Professor of Medicine, Stanford Prevention Research Center, Stanford University School of Medicine; Monterey County Health Department, Salinas, California; Monterey County Health Department, Salinas, California; Monterey County Health Department, Salinas, California

## Abstract

**Background:**

In 2003, the Monterey County Health Department, serving Salinas, California, was awarded one of 12 grants from the Steps to a HealthierUS Program to implement a 5-year, multiple-intervention community approach to reduce diabetes, asthma, and obesity. National adult and youth surveys to assess long-term outcomes are required by all Steps sites; however, site-specific surveys to assess intermediate outcomes are not required.

**Context:**

Salinas is a medically underserved community of primarily Mexican American residents with high obesity rates and other poor health outcomes. The health department's Steps program has partnered with traditional organizations such as schools, senior centers, clinics, and faith-based organizations as well as novel organizations such as employers of agricultural workers and owners of taquerias.

**Methods:**

The health department and the Stanford Prevention Research Center developed new site-specific, community-focused partner surveys to assess intermediate outcomes to augment the nationally mandated surveys. These site-specific surveys will evaluate changes in organizational practices, policies, or both following the socioecological model, specifically the Spectrum of Prevention.

**Consequences:**

Our site-specific partner surveys helped to 1) identify promising new partners, select initial partners from neighborhoods with the greatest financial need, and identify potentially successful community approaches; and 2) provide data for evaluating intermediate outcomes matched to national long-term outcomes so that policy and organizational level changes could be assessed. These quantitative surveys also provide important context-specific qualitative data, identifying opportunities for strengthening community partnerships.

**Interpretation:**

Developing site-specific partner surveys in multisite intervention studies can provide important data to guide local program efforts and assess progress toward intermediate outcomes matched to long-term outcomes from nationally mandated surveys.

## Background

Public health in recent decades has focused on a socioecological approach that emphasizes social and cultural modifications to change health behaviors (1,2). This approach addresses multiple societal influences on individual behavior with coordinated, multicomponent programs. These programs often combine more traditional health promotion methods with widespread health education and environmental and policy-level change to influence behavior (3). An important aspect of the socioecological approach includes developing novel partnerships to access or focus on smaller, at-risk populations often overlooked by existing approaches (4).

The socioecological approach helps health professionals develop improved models that encompass the complexity of multiple program components and interconnected intervention strategies that encourage sustainable systems change (5). The Spectrum of Prevention (the Spectrum) (6)The socioecological approach helps health professionals develop improved models that encompass the complexity of multiple program components and interconnected intervention strategies that encourage sustainable systems change (5). The Spectrum of Prevention (the Spectrum) (6) is one socioecological model used by many local and federal public health programs applying community-based approaches. The Spectrum is composed of six interrelated action levels: 1) strengthening individual knowledge and skills, 2) promoting community education, 3) educating providers, 4) fostering coalitions and networks, 5) changing organizational practices, and 6) influencing policy and legislation (6). This framework is especially useful for evaluating outcomes of community-based approaches that involve many interrelated and complex programs and partners at multiple levels of implementation.

In 2003, the Monterey County Health Department in California was one of 12 initial sites selected to receive funding from the Steps to a HealthierUS (Steps) cooperative agreement program. This funding is being used to implement a 5-year, multiple-intervention community approach to reduce the burden of diabetes, asthma, and obesity. Evaluation is a substantial component of the Steps program's national and community-level investment. All Steps program sites annually implement mandated Behavioral Risk Factor Surveillance System (BRFSS) surveys and Youth Risk Behavior Surveillance System (YRBSS) surveys. In Salinas, these two surveys have been modified to delete questions unrelated to the interventions and to add more detailed sociodemographic questions, so evaluators can determine whether outcomes differ among population subgroups. All Steps sites use a national logic model and guidance documents to assess community-level progress toward a set of long-term outcome measures (6). These surveys assess long-term outcomes; they do not focus on more intermediate outcomes related to site-specific intervention efforts.

At the start of the program, the health department used the Spectrum framework to plan the Steps to a Healthier Salinas program interventions. This plan included developing partnerships with traditional partners such as schools, senior centers, clinics, and faith-based organizations, as well as with novel partners such as employers of agricultural workers and owners of taquerias. The aims are to 1) build on and expand a set of past and current programs related to preventing obesity, diabetes, and asthma by increasing physical activity and good nutrition, and by decreasing smoking; and 2) emphasize disease management through more effective referral and treatment systems in secondary prevention programs. Plans are guided by evidence-based and cost-effective strategies and are tailored to the health literacy, linguistic, and cultural needs of Hispanics and other groups in the community.

To assess change at all levels of the Spectrum and to specifically assess progress toward intermediate outcomes linked to long-term outcomes in a local logic model, the Salinas program developed site-specific partner surveys to gauge organizational practices, policies, or both as implemented by community partners. This paper describes 1) the development and implementation of these surveys, 2) how the surveys were used to prioritize recruitment of community partners and to identify potentially successful community approaches, and 3) how these surveys will be used to evaluate intermediate outcomes matched to mandated national long-term outcomes.

## Context

Salinas, California, with 157,000 residents in 2005 (7), is a medically underserved community known for its international agribusiness industry. Hispanics make up 64% of the population, among whom 88% are of Mexican descent and 15% are agricultural workers (8). Random surveys of community and agricultural labor camp samples, conducted in 1990 and 2000, showed that most residents were young, had low education levels, spoke a language other than English at home, and had lived in the United States 10 years or more (9). The surveys also showed that from 1990 to 2000, many residents were at continuing or increased risk for chronic disease; for example, the prevalence of obesity increased by 47% (among community samples) and by 91% (among labor camp samples) (8). In 2000, residents also reported a low consumption of fruits and vegetables and a high consumption of fried foods.

The Spectrum was used as a unifying organizational framework for linking Steps interventions with traditional and novel community partners. Interventions are numerous and specific to partner organizations. The interventions include activities such as developing walking clubs; providing training on obesity, asthma, and diabetes in partnership with community members; and facilitating the adoption of exercise and nutrition policies. The Spectrum was also used to identify gaps in the proposed national evaluation framework, leading the evaluation team to develop site-specific partner surveys to capture intermediate and long-term outcomes related to organizational change that may result from specific intervention efforts in Salinas.

## Methods

### Development of site-specific surveys

We in the health department collaborated with the Stanford Prevention Research Center to develop a local program logic model that incorporated resources and activities or strategies. The model linked proposed interventions with expected local intermediate and national long-term outcomes. We then used telephone directories and county agency lists to create a comprehensive list of potential partners that included all schools, senior centers, clinics, faith-based organizations, employers of agricultural workers, and owners of taquerias with a Salinas address. We developed five site-specific partner surveys to ensure that intermediate outcomes were assessed. Other tracking mechanisms not reported here were developed for assessing change in community clinics. The five surveys complemented the mandated national annual BRFSS and biennial YRBSS. The surveys were administered at baseline in year 1 (2004) before the start of the interventions and will be repeated in year 3 at the midpoint of the interventions and in year 5 at the conclusion of the interventions.

When developing the site-specific surveys, we reviewed the literature to identify existing survey tools that assess similar types of programs and interventions, and when they were available and appropriate, we modified standardized survey questions to better represent the Salinas community approaches and outcomes. Most questions on the surveys were new and focused on organizational and policy areas: 1) healthy food policies, 2) healthy exercise policies, 3) smoke-free policies, 4) healthy food marketing strategies, and 5) wellness programs. The surveys also contained questions about the size of the organizations and profiles of their members.

A senior epidemiologist oversaw all survey development. Trained health department survey workers conducted the in-person and telephone administration of surveys. For surveys that were mailed and not returned promptly, survey workers made telephone calls until a completed survey was returned, participation was declined, or repeated telephone calls were not returned. Surveys were assessed for face validity by the senior epidemiologist, several chronic disease intervention specialists, and a biostatistician, and were revised on the basis of their suggestions. Surveys took 10 to 20 minutes to complete and were administered in English, except for a few surveys that were completed in Spanish during telephone call-backs to faith-based organizations. Other details of the five site-specific surveys follow:

- School districts (n = 4). A survey on the health policies proposed for Salinas school districts was developed and administered by telephone to school district superintendents or their health coordinators or nurses.
- Senior centers (n = 5). A survey on the health policies and health-related programs proposed for Salinas senior centers was administered by telephone to a central health program coordinator.
- Taquerias (n = 34). A survey on the interventions proposed for taquerias was completed by visiting all Salinas taquerias listed with the health department as possessing current food serving licenses. A taqueria was defined as an establishment serving Mexican-style dishes (e.g., burritos, tacos), where customers placed their orders at a counter, the menu was generally posted on the wall behind the counter, and customers' orders were called out or brought to them. All but a few survey questions could be answered by assessing the posted restaurant menu, the area around the counter, and the seating area. For the few questions related to taqueria health-related activities and customer options for menu items, the survey workers asked the questions of the owner (if present) or the on-site shift supervisor or manager.
- Employers of agricultural workers (n = 26). A survey on the health policies and health-related programs proposed for employers of agricultural workers was mailed to a list of employers with more than 100 employees. The list of companies was developed using the telephone directory. The human resources office of each company was called to confirm the number of workers employed and to identify companies that may have been missed from the telephone directory compilation.
- Faith-based organizations (n = 70). A survey on the health policies and interventions proposed for Salinas faith-based organizations was mailed to a list developed using the telephone directory. A faith-based organization was defined as an organization or group at a physical address that held regular faith-oriented meetings.

### Selecting initial partners and analytic approach

Although we hope that all partners will eventually adopt measures related to improving intermediate and longer-term outcomes, limited staff and resources required that we prioritize which organizations and businesses to partner with during the initial years of the interventions. To assist with selecting initial partners and ensure that partners were selected from neighborhoods with the greatest financial need, we divided the 25 Salinas census tracts into three groups, based on annual family income: low income (the bottom 25% of family income distribution; range of average incomes, $25,145–$34,112), moderate income (the middle 50%; range, $34,162–$53,500), and high income (the top 25%; range, $54,571–$83,123). The distribution of potential community partners among the three income categories was assessed with analysis of variance and chi-square analysis. A matrix of partner groups, intervention areas, and outcomes was developed to ensure that the surveys encompassed all intervention areas and outcomes.

## Consequences

Significant geographic stratification of sociodemographic characteristics existed within Salinas by level of census tract income (Table 1). Enumeration of organizations indicated a large base of potential partners with which to develop our interventions on policy and organization change. When we reviewed the geographic distribution of our potential community partners, we found that taquerias, faith-based organizations, and employers of agricultural workers (but not senior centers) were concentrated in the lower-income census tracts where individuals in greatest need reside. School districts were not included in this analysis because they overlapped census tracts.

Response rates to the five site-specific partner surveys varied from 100% for school districts, senior centers, and taquerias to 70% for agricultural worker employers and 69% for faith-based organizations. For agricultural worker employers, five companies declined to participate because of seasonal time constraints, and no response was obtained from six companies despite repeated callbacks. For faith-based organizations, 23 refused to participate and 9 were never reached at the listed phone number, despite repeated attempts.

Table 2 presents a summary of the content of the five site-specific surveys and how the surveys will track local intermediate outcomes matched to national long-term outcomes. Survey questions match the five intervention areas and corresponding local intermediate outcomes that can be linked to national long-term outcomes. This linkage will enable the local evaluation team to produce analytic reports for program use. For example, outcome data can be presented by type of organization and intervention area (e.g., changes in healthy food policies in schools and faith-based organizations) and posted on the program Web site for community stakeholders.

The site-specific surveys, administered before the start of the interventions, also contributed to targeting program planning. The administration of the surveys served as the first contact between the health department staff and potential partners, when survey workers introduced the program and asked for permission to conduct the survey. Information about the potential partner helped health department staff decide which organizations should be contacted next by the intervention staff. For example, for employers of agricultural workers and for faith-based organizations, data on the size of the organizations as well as their geographic distribution were useful in allocating staff resources for developing partnerships and interventions. In addition, the baseline surveys helped identify organization-specific concerns about proposed community approaches. For example, we found that taqueria owners initially mistrusted the health department because taquerias' past involvement with the health department mainly related to obtaining operating permits. This mistrust was partially overcome when survey workers developed a personal rapport with the owners and provided them with health information about diseases common to families and customers, in particular diabetes and obesity. As a result of these conversations, community approaches for taquerias were modified to include developing and promoting healthy food choices for diabetic patrons of the taquerias. We also identified organization-specific concerns from agricultural worker employers related to the timing of our planned interventions. We learned that because of the seasonality of their work, agricultural employers preferred to begin their participation during the off-season (November through March). We also learned that faith-based organizations were often reluctant to complete surveys because of concerns about the involvement of the government, as represented by the health department, in their operations. As a result, program staff modified their plans to focus on developing relationships with faith-based organizations before introducing intervention programs.

## Interpretation

Evaluation of the Steps to a Healthier Salinas Program's success toward creating sustainable community and environmental change within a 5-year period is a seemingly difficult goal. This is true for evaluating long-term outcomes only (e.g., changes in overweight and obesity) that are the focus of the national surveys, and for evaluating the intermediate outcomes that are the focus of our site-specific partner surveys (e.g., changes in food policies adopted by faith-based organizations).

Developing site-specific surveys has been important for several reasons. First, the surveys of all potential community partners helped us to identify promising new partners, select initial partners who were from neighborhoods with the greatest financial need, and learn about potentially successful community approaches. Second, the surveys will provide data for gauging progress toward intermediate, local outcomes that are more likely than long-term outcomes to occur in the short time frame of the intervention. Data on intermediate outcomes, linked to interventions, may also help in obtaining continued funding after the completion of the grant. Third, the surveys served to assess how the health department and organizations can work together to identify areas of need and potential challenges at the organization level. The benefits of these site-specific surveys relate to the importance of the social environment in defining issues to be addressed and to how a community defines the boundaries of possible action and change when implementing comprehensive community-based approaches (10). Thus, although the surveys focused on outcomes, they also helped guide the intervention staff in areas of qualitative project management. This process included developing new community partnerships and providing insight into the merit of various strategies during program development and potentially reducing staff time needed to establish program direction. The surveys also provided opportunities for program staff to identify areas for bridging social capital within and between traditional and novel partners. Identification of community forces, such as areas of resistance that may affect desired outcomes, has also been proposed as another important next step in understanding change in the context of multi-intervention programs (11).

We developed our site-specific partner surveys to augment the mandated national surveys and to help assess changing organizational practices or policies using the Spectrum framework. Questions on these surveys match intervention areas and corresponding local intermediate outcomes that are matched to national long-term outcomes. Such careful planning provides important quantitative evaluation data for the Spectrum levels of organizational and policy change. Interestingly, the site-specific surveys also provide valuable qualitative data that have informed areas of need for community education and have identified opportunities for developing or strengthening community partnerships, two other important levels of the Spectrum.

The community partner surveys for the Steps to a Healthier Salinas serve as a case study for developing a methodology to evaluate a multiple-intervention program in a community. These surveys will gauge progress toward intermediate outcomes that are matched to long-term outcomes. The documentation of site-specific intermediate outcomes are valuable to our community partners, health professionals, and community members as they work together to enhance the health of the Salinas population.

## Figures and Tables

**Table 1:** Sociodemographic Profile^a^ and Potential Community Partners for Salinas, California, by Income Level^b^, Steps to a Healthier Salinas, 2004

	Salinas	Low Income	Moderate Income	High Income	*P* Value
**Sociodemographic characteristic (2000)^c^ **					
Population, no.	151,060	6,752^d^	5,517^d^	5,694^d^	.75
Aged <18 y, %	32	36	32	28	.14
Median annual family income, $	43,720	30,817	44,302	63,107	<.001
Hispanic or Latino, %	64	83	57	45	.01
White, non-Hispanic, %	24	12	32	36	.04
Less than high school education, %	32	65	37	28	.01
College graduate, %	12	5	14	20	.01
Foreign-born, %	35	46	30	24	.01
Speak language other than English at home, %	60	74	53	46	.05
Unemployed, %	7	10	7	3	<.001
Income below federal poverty guidelines, %	17	24	11	6	<.001
Single female-headed households in poverty, %	26	38	26	18	.06
Moved in last 5 years, %	58	52	53	55	.85
**Community partners (2004)^e^ **					
Senior centers, no.	5	2	1	2	.43
Taquerias, no.	34	21	8	5	.01
Faith-based organizations, no.	70	32	24	14	.04
Employers of agricultural workers, no.	26	19	4	3	.01

a U.S. Census 2000.

b Low income was defined as the bottom 25% of the family annual income distribution (range, $25,145–$34,112); moderate income, the middle 50% (range, $34,162–$53,500); and high income, the top 25% (range: $54,571–$83,123).

c *P* value from a one-way analysis of variance between groups.

d Average population of census tracts in each income category.

e School districts are not listed because they overlap census tracts. *P* value from chi-square test.

**Table 2:** Community Partners, Intervention Areas, and Organizational-Level Measures in Relation to Intermediate Local and Long-Term National Outcomes, Steps to a Healthier Salinas, 2004

Partner/Intervention Area	Intervention Measure	Outcome Level	
		Intermediate Local	Long-Term National
**School districts **			
Healthy food and exercise policies	**Increased percentage of school districts with policies about**		
	Access to school yards and/or playgrounds for the community after school hours	A, C	3, 6, 7, 8, 10
	Access to inside school gyms for the community after school hours	A, C	3, 6, 7, 8, 10
	Healthy food being served or offered at school activities (e.g., sports events)	A, B	1, 6, 7, 8, 10
	Healthy food being served or offered in the cafeteria or snack bar(s)	A	1, 6, 7, 8, 10
	Healthy drinks in the school vending machine(s)	A, C	3, 6, 7, 8, 10
	Physical activity for students during school hours	A	1, 9, 10
Smoke-free policies	Increased percentage of school districts with no smoking within 200 feet of campus boundaries	A	1, 9, 10
**Senior centers**			
Healthy food and exercise policies	**Increased percentage of senior centers offering in past year**		
	Nutrition education events (e.g., cooking class, speaker about healthy foods)	A, B	1, 3, 7, 8, 10
	Exercise or physical activity events (e.g., dance, stretching and flexibility class, walking club)	A, C	1, 3, 7, 8, 10
	Sports events (e.g., lawn bowling, softball)	A, D	1, 3, 7, 8, 10
	Health fairs	A, B, C, D	1, 3, 7, 8, 10
	Screenings for diabetes	A, D	2, 5, 9, 10
	Screenings for asthma	A, D	2, 5, 9, 10
**Taquerias**			
Healthy food marketing strategies	Increased percentage of taquerias offering healthy food options on menu	A, B	1, 3, 7, 8, 10
Smoke-free policies	Increased percentage of taquerias with smoke-free entryways (20-ft radius)	A	1, 9, 10
**Faith-based organizations**			
Healthy food policies	**Increased percentage of faith-based organizations**		
	Distributing health information to congregations in sermons, bulletins, and/or newsletters in past year	A, B, C, D	1, 2, 3, 4, 5, 6, 7, 8, 9, 10
	Serving healthy food before and/or after services and youth group meetings in past year	A, B	1, 3, 7, 8, 10
	Hosting health-related activities in past year	A, B, C, D	1, 2, 3, 4, 5, 6, 7, 8, 9, 10
	With health-related food and exercise policies	A, B, C	1, 3, 7, 8, 10
Smoke-free policies	Increased percentage of faith-based organizations with smoke-free buildings and grounds	A	1, 9, 10
**Employers of agricultural workers**			
Wellness programs	**Increased percentage of employers**		
	Distributing health information with employee paychecks in past year	A, B, C, D	1, 2, 3, 4, 5, 6, 7, 8, 9, 10
	Hosting health-related activities in past year	A, B, C, D	1, 2, 3, 4, 5, 6, 7, 8, 9, 10
	With workplace policies for healthy food and exercise	A, B, C	1, 3, 7, 8, 10
Smoke-free policies	Increased percentage of employers with smoke-free entryways (20-ft radius)	A	1, 9, 10

a Intermediate local outcomes:
Implement organizational policies and changes related to physical activity, health services, health education, and tobacco use at community partner sites. Increase consumption of fruits, vegetables, and grains. Increase levels of moderate and vigorous physical activity. Develop referral systems and comprehensive case management programs for patients to improve appropriate self-care for obesity, diabetes, and asthma.

b Long-term national outcomes:
Increase knowledge and awareness about healthy behaviors such as physical activity, healthful eating, and avoiding tobacco use. Increase knowledge about getting appropriate preventive screenings. Increase physical activity and healthful eating among children and adults. Improve access to and quality of clinical services for asthma, diabetes, and tobacco cessation. Increase identification of people with prediabetes and diabetes. Improve self-management of asthma and diabetes. Measurable improvements in physical activity, healthful eating, and tobacco use. Slow the upward trend of overweight and obesity in Steps communities. Reduce hospitalizations due to asthma exacerbations and diabetes complications. Improve health-related quality of life.
